# Structure-Preserving Imitation Learning With Delayed Reward: An Evaluation Within the RoboCup Soccer 2D Simulation Environment

**DOI:** 10.3389/frobt.2020.00123

**Published:** 2020-09-16

**Authors:** Quang Dang Nguyen, Mikhail Prokopenko

**Affiliations:** Centre for Complex Systems, Faculty of Engineering, University of Sydney, Sydney, NSW, Australia

**Keywords:** deep learning, imitation learning, end-to-end learning, learning with structure preservation, learning with delayed reward, deep reinforcement learning

## Abstract

We describe and evaluate a neural network-based architecture aimed to imitate and improve the performance of a fully autonomous soccer team in RoboCup Soccer 2D Simulation environment. The approach utilizes deep Q-network architecture for action determination and a deep neural network for parameter learning. The proposed solution is shown to be feasible for replacing a selected behavioral module in a well-established RoboCup base team, *Gliders2d*, in which behavioral modules have been evolved with human experts in the loop. Furthermore, we introduce an additional performance-correlated signal (a delayed reward signal), enabling a search for local maxima during a training phase. The extension is compared against a known benchmark. Finally, we investigate the extent to which preserving the structure of expert-designed behaviors affects the performance of a neural network-based solution.

## 1. Introduction

Deep Learning provided a major breakthrough in Artificial Intelligence (AI). Due to high capability of generalization after training on a large number of samples, a neural network is able to learn complex multivariate functions, both linear and non-linear. A deep learning architecture uses multiple layers of transforming an input *x* ∈ *X*, to attain the best representation *T*(*x*) in making a prediction ŷ close to a target output *y* ∈ *Y*, by minimizing the loss function *L*(ŷ, *y*). Advances in deep learning have been followed by multiple successes in robotics (Atkeson and Schaal, 1997; Thrun et al., 2006; Lenz et al., 2015; Gu et al., 2017).

One of the important problems in robotics is to determine situation-based and goal-directed actions for agents. This problem has been addressed by deep learning algorithms developed along two promising directions: Imitation Learning (IL) and Reinforcement Learning (RLs). Learning merely from a feedback signal has been the ultimate goal of robotics and AI researchers for a long time. However, due to the sparsity of feedback and long decision horizons, there is still a limited number of successful studies, especially applied for practical problems. A way to deal with these difficulties is offered by IL. In this scheme, the agents try to mimic existing expert-designed systems using a large number of demonstrations, hoping to replicate the expert systems' performance. One typical approach is to directly learn the mapping between the environment states (*inputs*) and the targeted actions (*outputs*) by using supervised learning. Classical examples include inductive learning (Sammut et al., 1992), learning by watching (Kuniyoshi et al., 1994), and learning from demonstrations (Amit and Mataric, 2002). A remaining challenge of these standard IL solutions is how to ensure that performance of the learned action policy matches the expert system's performance consistently.

Reinforcement Learning aims to select rational actions for agents under the assumption that a reward signal is available to guide agents' decisions. Driven by the goal of maximizing a reward accumulated from a valid sequence of actions, RL addresses the need to both exploit and explore the problem space, in order to find an optimal solution “from scratch”, i.e., without labeled data. Successful examples include Deep Q-Learning (DQN) (Mnih et al., 2015), Deep Deterministic Policy Gradient (DDPG) (Lillicrap et al., 2016), Trust Region Policy Optimization (TRPO) (Schulman et al., 2015), Proximal Policy Optimization (PPO) (Schulman et al., 2017). However, a real challenge to RL approaches is the poor performance in the learning stage, normally requiring a large number of training steps to reach an acceptable performance policy. This is not easily applicable in real-world tasks when the agents have to endure consequences of their explorative actions in real time (Hester et al., 2018). The requirement to achieve an acceptable performance from the beginning, while still exploring how to improve agent's policy, remains a challenge. Hester et al. (2018) study addresses this problem with the introduction of Deep Q-Learning from Demonstrations. Other approaches, also related to the policy learning using experts' demonstrations, include methods developed by Chemali and Lazaric (2015), Subramanian et al. (2016), Brys et al. (2015), Cederborg et al. (2015), and Kim et al. (2013).

Addressing the problem of action policy learning in realistic environments, we focus on developing a learning scheme that takes advantage of both IL and RL. We aim to utilize the availability of expert's knowledge (e.g., demonstrations) and guide the learning by an additional delayed reward signal correlated with the final performance. This learning scheme is assessed in a multi-agent environment with the objective to replace and improve an expert-designed behavioral module. Unlike typical RL environments, we make no assumption on the availability of standard reward signals and the continuity of the state transitions: that is, the reward signals can be sparse and not present in the recorded demonstrations. Additionally, in contrast to the work of Hester et al. (2018) which requires both pre-training (offline) phase with demonstrations and training (online) phase with the interactions with the actual environment, we focus on offline learning with the expectation to have a strong team performance from the very beginning. The proposed solution provides a framework to guide agents' learning with an artificial imitation signal and delayed reward signal, in order to (i) minimize the difference between the learned policy and the target policy (or expert's policy) of a single agent, as well as (ii) maximize relevant team-level rewards which correlate with the final team performance.

We apply our learning framework to the multi-agent environment of RoboCup Soccer 2D Simulation (RCSS). This environment models multiple features from the real-world (robotic) soccer, such as distributed control, partial observation induced by noise, highly-changing and uncertain dynamics, and heterogeneity of agent types (Noda et al., 1998). While several machine learning approaches have been applied to multi-agent systems (MAS), including RoboCup soccer, the topic continues to attract vigorous interest. Furthermore, dominant approaches to RCSS are still based on well-designed behavioral decomposition and modularization. Recent successes of champion teams, WrightEagle (2006, 2009, 2011, 2013, 2014, 2015), Helios (2010, 2012, 2017, 2018), Gliders (2016), and Fractals (2019), with the last three teams based on *Agent2d* architecture, have proved the advantage of expert-designed structures with a partial, rather than an end-to-end learning architecture (Bai et al., 2015; Prokopenko and Wang, 2017, 2019a; Akiyama et al., 2019). The possibility of replacing an entire system or behavioral modules by a single neural network, i.e., end-to-end learning, or replacing the modules by multiple neural networks, following an original code structure, i.e., learning with structure preservation, remains a subject of research. This paper directly addresses this question, aiming to find an effective way to replace expert-designed modules with a neural architecture.

In short, we propose an RL-based imitation learning scheme utilizing both the expert's demonstrations and the delayed reward signal, artificially created and highly correlated with the final performance. Furthermore, we investigate the effectiveness of the end-to-end learning and the partial learning preserving a structure of expert-designed systems, particularly, the ones which proved their benchmark performance. We focus on a case study of learning defensive behaviors using the base team *Gliders2d* (Prokopenko and Wang, 2019a) in RCSS.

Section 2 lays the foundations for our proposed framework. Section 3 concentrates on modeling the problem and details our implementation for both (i) end-to-end imitation learning and (ii) structure-preserving imitation learning with delayed reward signal. Finally, section 4 describes experimental results, and discusses the proposed framework and its applications.

## 2. Background and Framework

In this section, we provide background and describe related works. Firstly, we introduce RCSS and popular MAS architectures developed in this environment. Then we briefly summarize modern approaches to imitation learning and reinforcement learning. Finally, we describe Deep Q-Learning, a specific RL algorithm, and its improved version.

### 2.1. RoboCup Soccer 2D Simulation Environment

RCSS is a decentralized simulation environment (Kitano et al., 1997) which has been used as an important testbed for MAS and AI. It is designed to emphasize selected features such as real-time actions, limited communication bandwidth, highly dynamic concurrent movements of autonomous agents, heterogeneity of different agent types, and adversarial multi-agent environment, comprising models to control the agents' vision, stamina, and other factors (Kitano et al., 1997, 1998; Noda et al., 1998; Stone and Veloso, 1999; Noda and Stone, 2003; Prokopenko and Wang, 2017). The objective of RCSS is to create an environment, based on football game rules, which can benchmark the performance of different multi-agent solutions and architectures (Kitano et al., 1997, 1998; Noda et al., 1998).

RCSS challenges have been handled by both expert-designed and machine learning approaches. On one hand, many approaches utilize human-selected features and expert-designed strategies, including Situation Based Strategic Positioning (Reis et al., 2001), multi-agent positioning mechanism (Akiyama and Noda, 2008), coordination system based on setplays (Mota and Reis, 2007), positioning based on Delaunay Triangulation (Akiyama and Noda, 2007), and Voronoi diagrams (Prokopenko and Wang, 2017). Others involve well-optimized defense and attack behaviors in popular code bases such as *Agent2d* (Akiyama and Nakashima, 2013) and *Gliders2d* (Prokopenko and Wang, 2019a,b). On the other hand, machine learning approaches have been applied in RCSS environment as well, e.g., a reinforcement learning approach (Riedmiller et al., 2001, 2008; Gabel et al., 2009), online planning with tree search method (Akiyama et al., 2012), and MAXQ value function decomposition for online planning (Bai et al., 2015).

In particular, team Helios improved their tree-search architecture with Support Vector Machine (SVM) used as a classifier to prune unsuccessful action sequences according to data collected and classified manually by experts (Hidehisa et al., 2017). Other improvements used SVMRank algorithm to provide better action evaluation (Akiyama et al., 2019). These improvements focused on improving existing modules of the expert-designed multi-agent architectures. Similarly, Prokopenko and Wang (2019a) replaced the fixed player types assignment as well as attacking tactics and offside trap behaviors in *Gliders2d* (version 1) by applying Dynamic Constraint Annealing method. In this paper, we propose a neural network-based design aimed to replace a specific expert-designed module for *defensive positioning* in *Gliders2d*. The new design aims to imitate, and then improve upon, the *Gliders2d*'s performance in defense.

**Figure 1:**
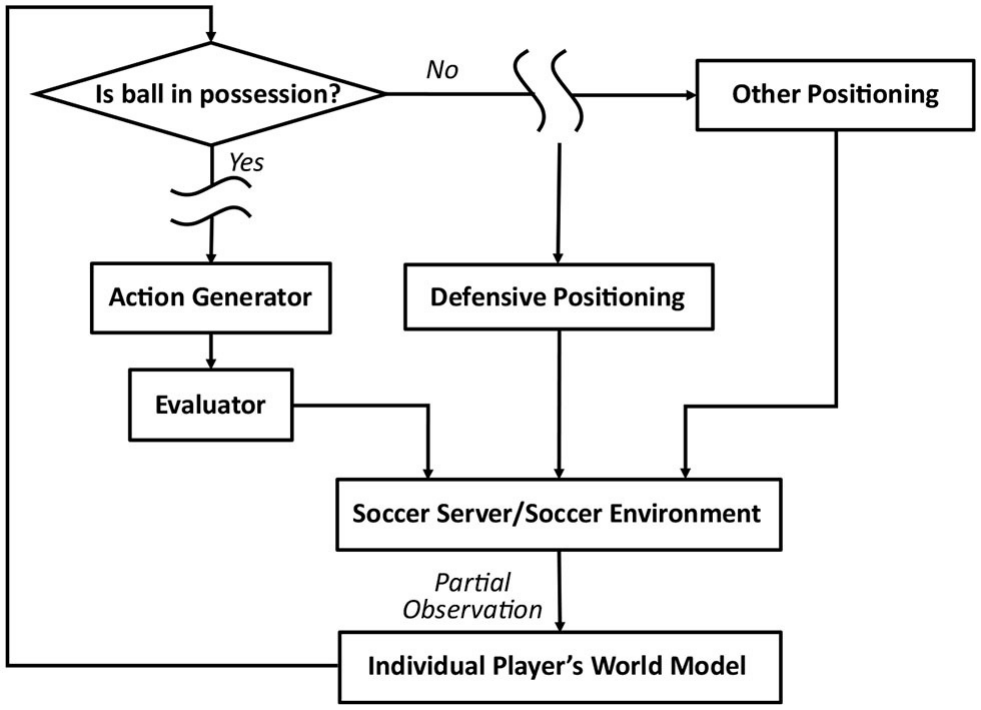
Overall framework of *Gliders2d*, including the module for *Defensive Positioning*.

We develop and evaluate a learning scheme, aiming to replace the expert-designed code for defensive behaviors, defined in the module for *defensive positioning*. The structure of this module, in context of the agent architecture, is summarized in Figure 1. Since RCSS is a decentralized multi-agent environment, each player's sensory observations received from RCSS server are partial (fragmented and latent), dependent on specific limitations of the player's vision. Other behavioral blocks in Figure 1 describe the simplified architecture for each player's controller, designed to select appropriate actions according to an individual world model, which is maintained by each player using partial sensory observations.

### 2.2. Imitation Learning and Multi-Objective Learning With Additional Delayed Reward Signal

In decentralized multi-agent problems, learning “from scratch” normally deals with very high dimensions over agents' actions and environment states, which are typically inaccessible. In practice, these challenges are often overcome by expert systems, heuristics, and/or rule-based approaches. As a result, learning to mimic, i.e., imitation learning (IL), and then improving upon these systems, has emerged as a reasonable approach, being more feasible than searching the entire space of states and actions.

Specifically, IL is a technique designed to mimic a policy by learning from recorded pairs of an existing system's states and corresponding actions, or demonstrations. It relies on the availability of a large number of demonstrations from the expert-designed system. The state feature set *S* and the possible action set *A* in IL are usually selected to match those in the original systems. In the simplest form, IL finds a mapping function *f* : *S* ↦ *A* to minimize the loss function $L={𝔼}_{s\sim p_{\pi }}\left[\ell \left(â=f\left(s\right),a\right)\right]$, where *p*_π_ is the state distribution under the expert-designed system's policy π, *s* ∈ *S* is the state following the distribution defined by policy π, i.e., *s* ~ *p*_π_, and *a* ∈ *A* is the corresponding action selected at state *s* by the expert system.

In a multi-agent context, we assume that *N* agents cooperate to realize a common goal, producing a training dataset *D* that contains demonstrations $d^{i}=\left\{d_{t}^{ij}=\left\{s_{t}^{i},a_{t}^{i}\right\}\right\}$, where *i* = 1…*N* is agent index, *j* is sample index, and *t* = 1…*T* is time index in a logged sequence. *T* can be a single time step for the case of contextual bandits or can be >1 for a temporal sequence of consecutive states and actions. In centralized multi-agent imitation learning, the loss function that needs to be minimized is given as follows (Le et al., 2017):

(1) $$\begin{array}{l} L_{centralised}=\underset{\vec{s}\sim p_{\vec{\pi }}}{𝔼}\left[\ell \left(\vec{\pi }\left(\vec{s}\right),{\vec{\pi }}_{l}\left(\vec{s}\right)\right)\right] \end{array}$$

where $\vec{s}\;\sim \;p_{\vec{\pi }}$ denotes the state distribution according to the expert system's joint policy $\vec{\pi }$ of all agents; $\vec{a}=\left\{a_{i}\right\}$ and $\vec{s}=\left\{s_{i}\right\}$ where *i* = 1…*N* are the set of all states and associated actions of all agents, and π_*l*_ denotes the policy learned from imitation learning process. The knowledge of $\vec{\pi }$, $\vec{\pi_{l}}$, and $\vec{s}$, is assumed to be accessible by all agents in a centralized manner.

For decentralized multi-agent imitation learning, e.g., in the RCSS environment, learning with independent agents is different. Specifically, the access to others' states and action policy is explicitly prohibited. The loss function merely depends on each agent's experience *d*^*i*^ including their own policy and observations. Equation (2) shows the general form of loss function for decentralized imitation learning:

(2) $$\begin{array}{l} L_{decentralized}^{i}=\underset{s\sim p_{\pi^{i}}}{𝔼}\left[\ell \left(\pi^{i}\left(s\right),\pi_{l}^{i}\left(s\right)\right)\right]\approx \underset{d_{ij}\in d_{i}}{\sum }\ell \left(d_{ij},{\hat{d}}_{ij}\right) \end{array}$$

where π^*i*^ and $\pi_{l}^{i}$ denote the policy of an expert-designed system, and the learned policy of agent *i* respectively. Using Monte Carlo method to approximate this loss function, we obtain a simpler version that can be calculated from collected demonstrations *D*. Relying on the generalization of neural networks, we train the policy network with an additional feature representing an agent index, which results in a loss function for training a single neural network used for all agents' policy:

(3) $$\begin{array}{l} L_{decentralized}\approx \overset{N}{\underset{i}{\sum }}\underset{d_{ij}\in d_{i}}{\sum }\ell \left(d_{ij},{\hat{d}}_{ij}\right) \end{array}$$

With the objective to minimize the loss function L, imitation learning sets the target to learn a new policy which has performance as close as possible to the expert-designed system's performance. The learning process follows gradient descent updating network parameters θ according to $\nabla_{\theta }L$.

Moreover, we aim to both mimic and improve the performance of learned policy in the decentralized environment. To achieve this target, we extend the problem of imitation learning and transform it into a reinforcement learning (RL) framework with an MDP, with 5-tuple *{State S, Action A, Reward R, Transition Probability P, Discount Rate* γ*}*. RL is a sub-category of Machine Learning which studies how an agent makes rational decisions in sequence, by exploring state and action space under the guidance of a reward signal. An agent following RL is supposed to take an action *a*_*t*_ = π(*s*_*t*_) according to RL-powered policy π, based on state information *s*_*t*_, in order to interact with the environment at time step *t*. As a consequence, in the next time step *t* + 1, it will receive the next observation about the next environment state *s*_*t*+1_ and reward from taken action *r*_*t*_. The ultimate goal of RL is to maximize the accumulated discounted return *G*_*t*_:

(4) $$\begin{array}{l} G_{t}=R_{t+1}+\gamma R_{t+2}+\gamma^{2}R_{t+3}+\cdots =\overset{T}{\underset{k=0}{\sum }}\gamma^{k}R_{t+k+1} \end{array}$$

where γ is the discount rate in the range [0, 1], and *R*_*t*_ denotes the reward at time step *t* (Sutton and Barto, 2018). The flexibility of RL framework in optimizing multiple objectives integrated within a reward signal addresses our need in both imitating and improving agents' policy with respect to existing expert-designed behavior.

**Figure 2:**
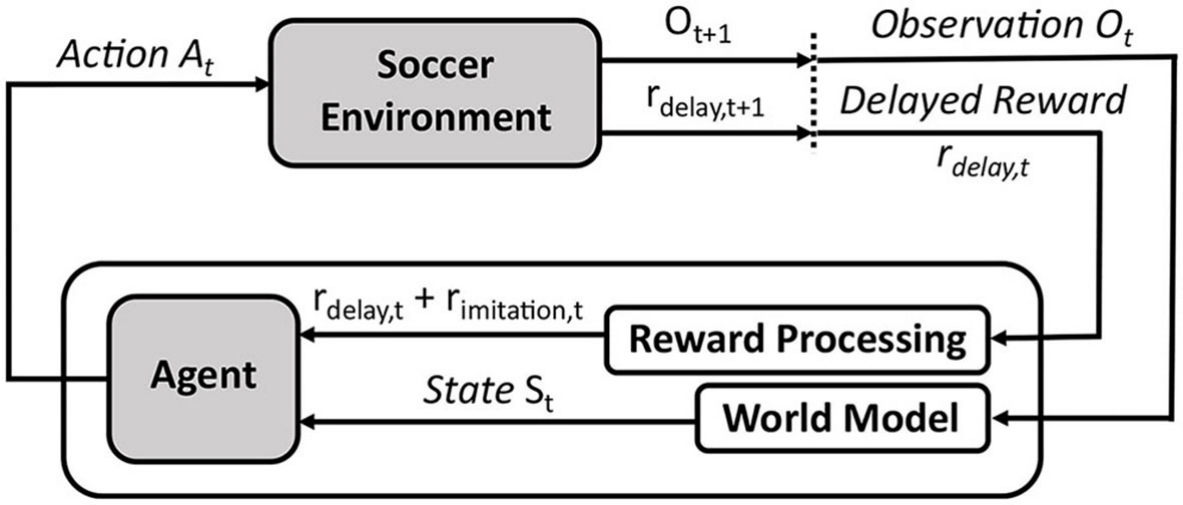
Overall RL framework to imitate and improve performance of *Gliders2d*'s defensive behaviors from demonstrations.

Within the RL framework, we can introduce different objectives to the system via a reward signal, and guide the training to maximize all of these targets. In our case of decentralized policy learning, these targets include (i) *minimizing imitation loss* to take advantage of the experts' demonstrations, and (ii) seeking *improvement in team performance* beyond the experts' demonstrations. We intend to introduce the imitation reward $r_{imitation}=-L_{decentralized}^{i}$ and a delayed reward signal *r*_*delay*_ which is highly-correlated with the team performance (for example, timing of scored or conceded goals—see section 3.2). Figure 2 illustrates the application of RL to our multi-objective problem in RCSS.

The idea to shape the reward function with the expert's demonstrations has been explored in previous studies, such as inverse reinforcement learning (Abbeel and Ng, 2004) and multi-motivation learning (Palm and Schwenker, 2019). However, implementation and evaluation of this idea in multi-agent environments are still lacking. In our study, we explicitly introduce a delayed reward signal enhancing the demonstrations used by RL framework, and evaluate this combination in a decentralized multi-agent environment.

### 2.3. Deep Q-Learning Algorithm

In this section, we detail our implementation of training the agent's policy, as shown in Figure 2. We propose an RL-based learning framework associated with the tasks of imitating and improving defensive behaviors in RCSS, by integrating Double DQN (Hasselt et al., 2016), as an RL algorithm, to train our neural networks for a decentralized action policy. The background of this algorithm is provided below.

In general, basic RL solutions involve a state-value function *V*_π_(*s*) = 𝔼_π_[*G*_*t*_|*S*_*t*_ = *s*] = 𝔼_π_[*R*_*t*+1_ + γ*G*_*t*+1_] and an action-value function *Q*_π_(*s, a*) = 𝔼_π_[*G*_*t*_|*S*_*t*_ = *s, A*_*t*_ = *a*] under policy π at state *s* ∈ *S* (Sutton and Barto, 2018). DQN algorithm (Mnih et al., 2013) defines its optimal policy by selecting the action having the most possibility to gain the highest return in the future, based on an optimal action-value function $Q^{*}\left(s,a\right)=\max_{\pi }Q_{\pi }\left(s,a\right)$ as follows:

(5) $$\begin{array}{l} Q^{*}\left(s,a\right)={𝔼}_{s^{\prime }\sim E}\left[r+\gamma \underset{a^{\prime }}{\max}Q^{*}\left(s^{\prime },a^{\prime }\right)|s,a\right] \end{array}$$

(6) $$\begin{array}{l} \pi \left(s\right)=\arg\underset{a\in A}{\max}Q^{*}\left(s,a\right) \end{array}$$

where $s^{\prime }\sim E$ denotes the next state distribution following the nature of the environment.

Furthermore, Mnih et al. (2013) used the function approximator method *Q*(*s, a*; θ) to approximate action-value function Q^*^(*s, a*) in Equations (5) and (6) by using a deep neural network implementation for the Q-networks. Equations (7) and (8) show the loss function and the gradient to be used to update Q-network parameters θ_*i*_ at iteration *i* during the value iteration process:

(7) $$\begin{array}{l} L_{i}\left(\theta_{i}\right)={𝔼}_{s,a\sim \rho \left(.\right)}\left[{\left(y_{i}-Q\left(s,a;\theta_{i}\right)\right)}^{2}\right] \end{array}$$

(8) $$\begin{array}{l} \nabla L_{i}^{Q}\left(\theta_{i}\right)={𝔼}_{s,a\sim \rho \left(.\right),s^{\prime }\sim E}[(r+\gamma Q\left(s^{\prime },\arg\underset{a}{\max}Q\left(s^{\prime },a;\theta_{i}\right);\theta_{i}\right)\\ \;-Q\left(s,a;\theta_{i}\right))\nabla_{\theta_{i}}Q\left(s,a;\theta_{i}\right)] \end{array}$$

where *s,a* ~ ρ(.) denotes the state and action distribution, following “the probability distribution over sequences *s* and actions *a*” (Mnih et al., 2013), and $s^{\prime }\sim E$ denotes the next state distribution.

In Equation (8), the same Q-network parameterized by θ_*i*_ is used for evaluation and action selection, which often leads to the issue of the “*overoptimism due to estimation errors”* (Hasselt et al., 2016). Therefore, instead of applying DQN, we select another version, Double DQN (Hasselt et al., 2016), to avoid this issue. Double DQN separates the use of Q-network for evaluation and action selection purposes by utilizing a system of two action-value neural networks: *Estimator* (parameterized by θ) and *Target* (parameterized by θ^−^), in order to perform a modified update step in Equation (9). The network parameters θ of Estimator are updated at every step, while target network parameters θ^−^ are periodically hard copied from θ.

(9) $$\begin{array}{l} \nabla L_{i}^{DQ}\left(\theta_{i}\right)={𝔼}_{s,a\sim \rho \left(.\right),s^{\prime }\sim E}[(r+\gamma Q\left(s^{\prime },\arg\underset{a}{\max}Q\left(s^{\prime },a;\theta_{i}\right);\theta_{i}^{-}\right)\\ \;-Q\left(s,a;\theta_{i}\right))\nabla_{\theta_{i}}Q\left(s,a;\theta_{i}\right)] \end{array}$$

## 3. RL-Based Approach to Learn Defense Behaviors in RCSS

### 3.1. Problem Modeling

**Figure 3:**
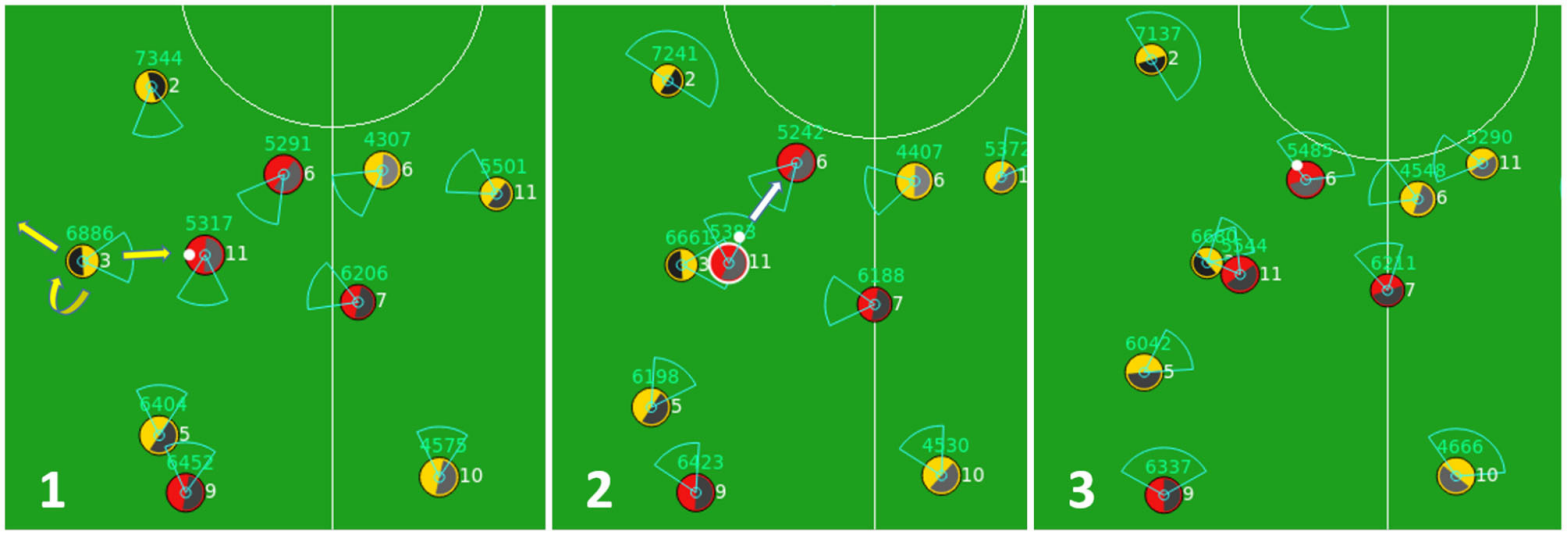
A sequence of three cycles when an agent (yellow team, number 3) activates a pressing behavior in cycle 1 that prevents the opponent (red team, number 11) from moving forward in cycle 2, and forces it to pass the ball back to its teammate in cycle 3.

Defensive behaviors of an agent implemented in baseline code *Gliders2d* are activated whenever the home team loses the ball. As shown in Figure 1, using the module for *defensive positioning* and the inputs from its world model updated based on most recent perceptions, the agent considers a selection of defensive behaviors, including tackling, intercepting, blocking, pressing, offside trapping, and so on, according to fixed pre-optimized rules. An illustration of defensive behaviors is given by Figure 3, showing how by processing this behavioral module, the agent activates a pressing behavior, which forces the opponent to pass the ball back, slowing the attack.

**Figure 4:**
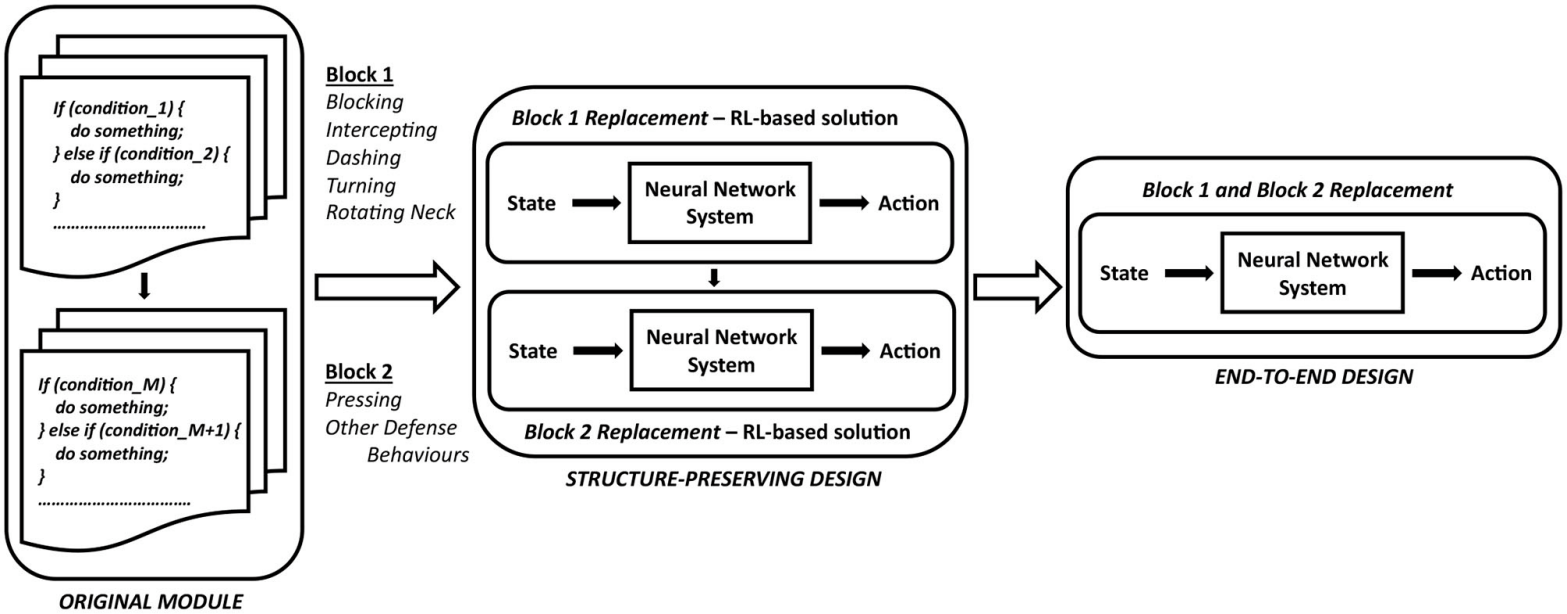
A general approach aiming to replace an expert-designed behavior by an RL-based neural network architecture.

Aiming to learn a policy for defensive behaviors from a baseline agent *Gliders2d*, we analyze the structure of the associated module and replace it by a neural network-based solution. This provides the benefits of integrating the knowledge about expert system's action policy, and enhancing team performance with the delayed reward signal. A general approach is described in Figure 4, showing how a pre-optimized code with a very large number of if-else rules, based on simplified MarliK code (Tavafi et al., 2012), is replaced by an RL-based neural network with the ability to learn from the expert system's demonstrations and reward signal.

The implementation of defensive behaviors in *Defensive Positioning* can be naturally divided into two sequential functional blocks, *Block 1* and *Block 2*, shown in Figure 4. We will investigate the effectiveness of changing such a structure from a expert-designed system to an RL-based neural network architecture, enhanced by the guiding signals from demonstrations and the reward information. This problem will be evaluated with an RL-based solution replacing *Block 1* and *Block 2*, following the structure-preserving architecture highlighted by Figure 4. Additionally, we examine the question of whether the structure of a expert-designed system is worth preserving, by analyzing the performance of this structure maintained with either two replacement blocks processed in sequence (the structure-preserving architecture shown in Figure 4), or a replaced structure comprising a single neural network managing a selection of all defensive behaviors (the end-to-end design, see Figure 4). The comparison between the structure-preserving learning and the end-to-end learning is carried out for our special case of learning defensive behaviors within a decentralized multi-agent environment, where expert-designed solutions have proved their dominance over several years of the RoboCup Simulation competitions.

**Figure 5:**
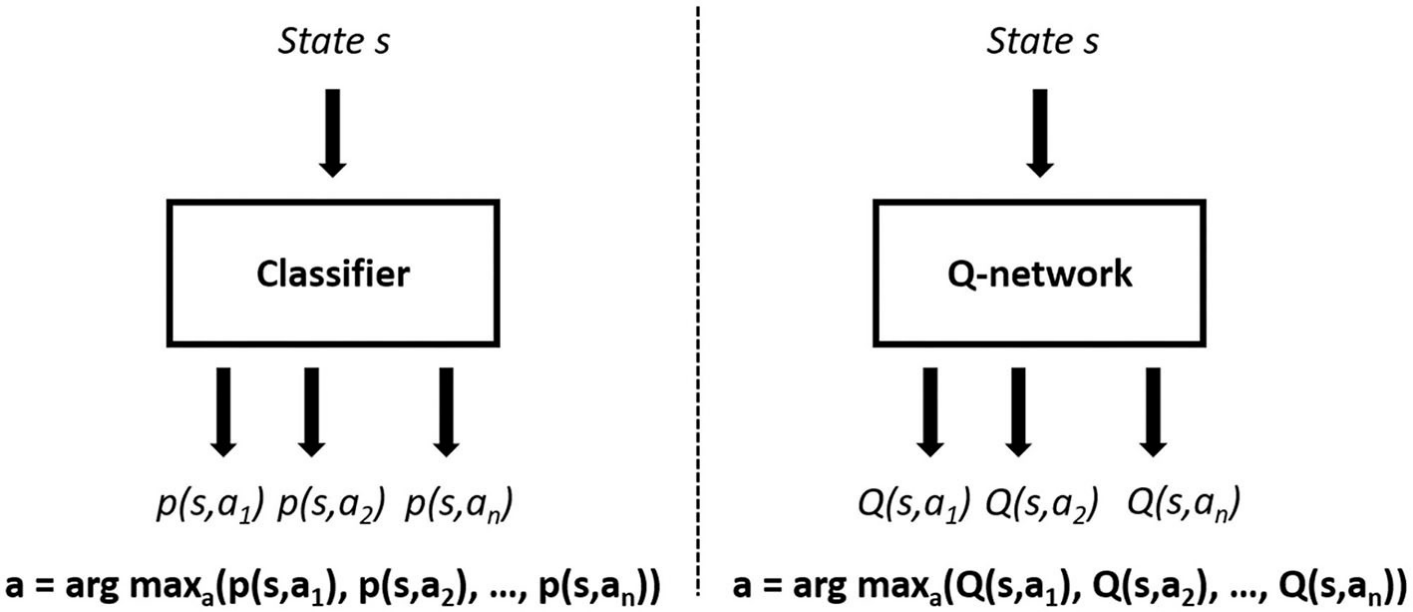
Classifier-based imitation learning framework **(left)** and Double DQN-based learning framework **(right)**.

Firstly, we model an RL-based learning scheme with Double DQN algorithm. As mentioned in section 2.1 (e.g., Figure 1), each agent selects an action according to their self-maintained world models. This expert-designed module reduces our scope of learning from a partially observable Markov decision process (POMDP) to a Markov decision process (MDP), which is suitable for the application of Double DQN to multi-agent environments. This RL-based learning approach also changes the problem of designing an n-class classifier (classical imitation learning) to the regression problem of estimating optimal future returns of all available actions, aiming to select the best action in every time step. This basic design leads to a difference between the architectures, as shown in Figure 5, where we use the action-value function *Q*(*s, a*) in selecting the optimal action, instead of the likelihood probability of actions according to historical demonstrations *p*(*s, a*). With Double DQN implementation, we introduce a reward signal, which is highly correlated with long-term team performance, as described in section 3.2.

Secondly, we define *f*_θ_1__ parameterized by θ_1_ as a general form of the neural network for *Block 1*, and *f*_θ_2__ parameterized by θ_2_ as a general form of the neural network for *Block 2*. We study the impact of structure preservation in learning defensive behaviors by examining two cases: (i) two networks *f*_θ_1__ and *f*_θ_2__ processed in sequence, following the design in *Gliders2d* (the structure-preserving architecture shown in Figure 4), and (ii) a single network for all actions *f*_θ_ (the end-to-end design shown in Figure 4).

### 3.2. RL-Based Learning Scheme for Action Selection in RCSS

**Figure 6:**
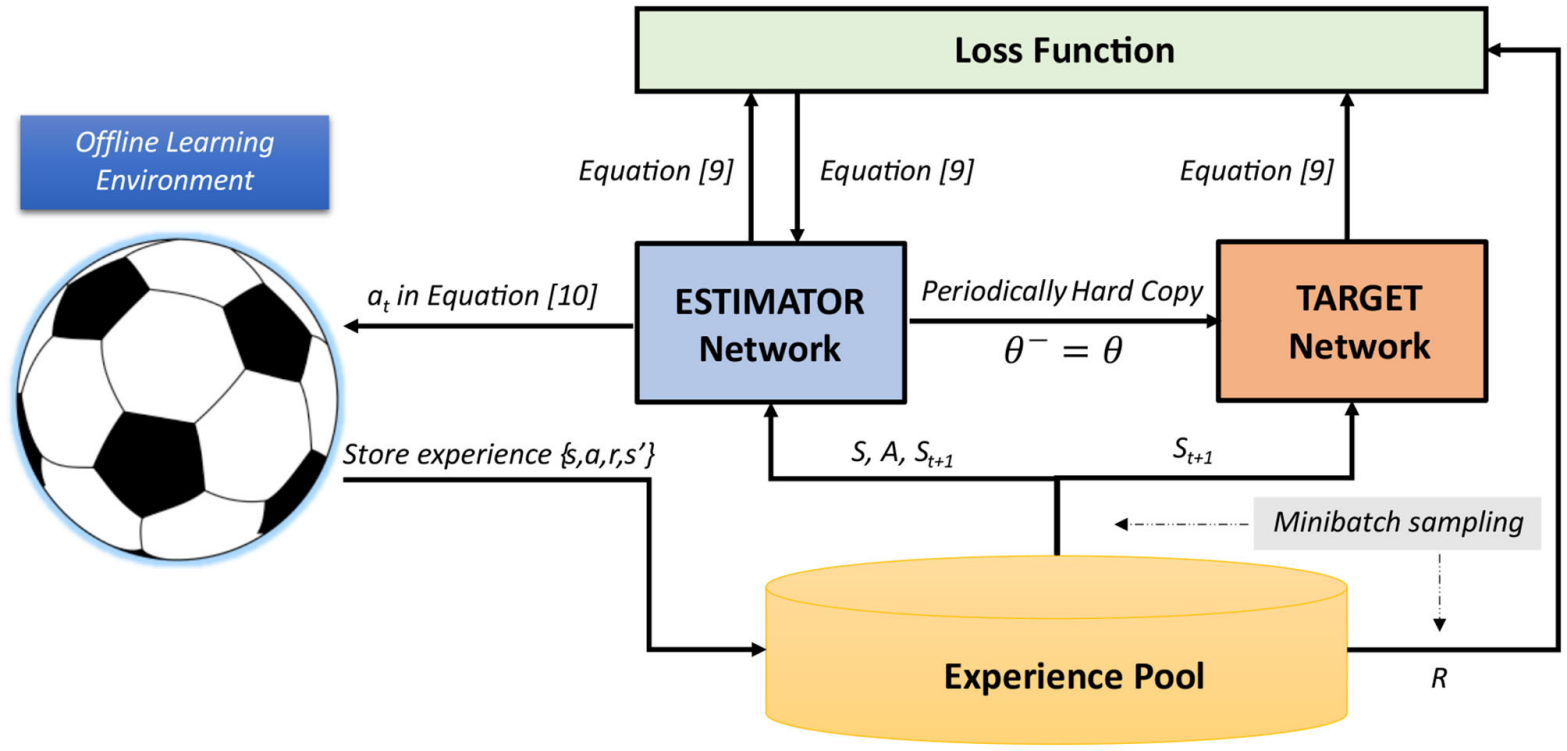
Overall RL-based learning scheme for action selection in RCSS.

Our new agent's behavioral module for defensive positioning relies on the action-value estimation of *Estimator* network trained with Double DQN algorithm, under the learning scheme provided in Figure 6. Components of this scheme include RL-styled *Environment, Experience Pool, Estimator* network, and *Target* network. We set the target to learn from offline demonstrations, which requires a setup of an artificial offline learning environment (OLE). We realize this task by logging the environment states and actions of all players in a sufficiently significant number of games, when their modules for defensive positioning are activated. The state and action sequences involved in our learning process are recorded from the traces produced by this behavioral module, and are not expected to be consecutive. That is, the actual reward signal can occur both in the recorded cycles (On cycles) and in the non-recorded cycles (Off cycles). We introduce a weighted reward signal back-propagated from the cycles in which the actual reward occurs. This signal is exponentially decreased from these cycles and is artificially applied to every recorded cycle. The final reward signal, used to guide the learning process, is the signal fusing the reward signal representing the goals and the signal rewarding the imitation of an expert-designed policy. Moreover, our delayed reward signal is used by all team members of a RoboCup team (differentiated by their unique team numbers which represent their field roles). This alleviates the credit assignment problem (Nguyen et al., 2018), and distinguishes our approach from the ones which use a standard reward signal.

Detailed steps for this learning scheme are specified below.

[1] A demonstration *d* is randomly selected from the logged data *D*. State information *s*_*t*_ is drawn from *d* and forwarded to the agent, while the reward information and the default system's action are kept within the scope of OLE to process the reward signal.

[2] *Estimator* network receives state *s*_*t*_ and predicts action-values *Q*(*s*_*t*_, *a*) of all possible actions *a* at state *s*_*t*_. The selected action is returned to *OLE* following ϵ-greedy exploration strategy (Equation 10).

(10) $$\begin{array}{l} a_{t}=\{\begin{array}{cc} \arg\max_{a}(\left\{Q\left(s_{t},a\right\}\right), & with\;probability\;1-\epsilon \\ randomaction, & with\;probability\;\epsilon \end{array} \end{array}$$

[3] Based on pre-selected demonstration from logged data, *OLE* calculates reward *r*_*t*_ and returns the next state *s*_*t*+1_, according to Equations (11) and (12), respectively. In Equation (11), λ_1_ and λ_2_ are the coefficients associated with the rewards from scored goals (goals of the home team) and conceded goals (goals of the opponent team). *T*_*scoring goal*_ and *T*_*conceding goal*_ are the earliest cycles that a goal is recorded after time cycle *t*. Index *demo* denotes the information extracted from logged demonstration, which is randomly selected by *OLE* from the beginning. The final experience of {*s*_*t*_, *a*_*t*_, *r*_*t*_, *s*_*t*+1_} is then stored in *Experience Pool*.

(11) $$\begin{array}{l} \begin{array}{l} r_{t}=r_{imitation,t}+r_{goal,t}\\ \;=\{\begin{array}{l} r_{imitation,t}+\lambda_{1}\times r_{\text{scoring goal},t}\\ +\lambda_{2}\times r_{\text{conceding goal},t},\;if\;a_{t}=a_{demo,t}\\ r_{imitation,t},\;if\;a_{t}\neq a_{demo,t} \end{array}\\ \;=\{\begin{array}{l} -L\left(a_{demo},a_{t}\right)+\lambda_{1}\times {\left(\tau \right)}^{T_{\text{scoring goal}}-t}\\ +\lambda_{2}\times {\left(\tau \right)}^{T_{\text{conceding goal}}-t},\;if\;a_{t}=a_{demo,t}\\ -L\left(a_{demo},a_{t}\right),\;if\;a_{t}\neq a_{demo,t} \end{array} \end{array} \end{array}$$

(12) $$\begin{array}{l} s_{t+1}=\{\begin{array}{l} s_{demo,t+1},\;if\;a_{t}=a_{demo,t}\\ END,\;if\;a_{t}\neq a_{demo,t} \end{array} \end{array}$$

[4] Next, we randomly select a minibatch of experiences from *Experience Pool*, in order to update *Estimator* network. Equation (9) is used to calculate gradient $\nabla L_{i}^{DQ}\left(\theta_{i}\right)$.

[5] After a fixed number of update steps for *Estimator* network, we hard-copy all *Estimator* network parameters θ to overwrite *Target* network parameters θ^−^.

In addition, because the sizes of action classes in *D* show a significant imbalance, we apply a special sampling technique to undersample the transitions from majority classes and oversample the transitions from minority classes. The algorithm and training hyper-parameters for RL-based learning are provided in Supplementary Material.

## 4. Experimental Results and Discussion

In this section, we present experimental results in order to (a) empirically prove the effectiveness of the proposed offline learning scheme with a delayed reward signal, and (b) compare the end-to-end design and the structure-preserving design in replacing expert-designed behavioral modules. The experiments are carried out to evaluate the neural network-based solution aimed to replace the expert-designed *defensive positioning* module. We evaluate our work by comparing our team performance versus a fixed benchmark opponent, i.e., team *YuShan*2018 (Cheng et al., 2018), in three test cases: (i) when replacing *Block 1* by an RL-based neural network, (ii) when replacing both *Block 1* and *Block 2* by two RL-based neural networks with the original structure preserved, and (iii) when replacing both Block 1 and Block 2 by a single RL-based neural network without preservation of the original structure. Test cases (i) and (ii) were aimed at objective (a), while the comparison between test cases (ii) and (iii) was targeted at objective (b). We note that this benchmark is representative of a sub-class of RCSS teams, implemented using *Agent2d* (Akiyama and Nakashima, 2013), being one of the strongest in this sub-class, having achieved a top six finish in 2018 world championship, and top four in 2019.

### 4.1. Replacement of Block 1 With RL-Based Neural Network

In this section, we describe results of learning to imitate (the first phase), and then applying imitation learning with the introduction of the delayed goal signal, by using the RL-based learning scheme (the second phase), as described in section 3.2. Two parameters, Blocking Position and Body Angle, have been learned independently, using DNN architecture.

*Block 1* in the module for *defensive positioning* is the controlling block, responsible for making a decision among six actions: Go to *Blocking Position* and Turn Neck (or *Goto+Neck*), Intercept and Turn Neck (or *Intercept+Neck*), Dash and Turn Neck (or *Dash+Neck*), Turn with *Body Angle*, and Turn Neck (or *Turn+Neck*), Turn Neck only (or *Neck*), or (as a fall-back option), Let *Block 2* make the decision (or *Block 2*). We used approximately four million transitions of defensive task to train the neural network-based replacement of *Block 1*. The specific percentages of records related to each action category are 28.2% for *Goto+Neck*, 8.2% for *Intercept+Neck*, 2.2% for *Dash+Neck*, 2.6% for *Turn+Neck*, 0.2% for *Neck*, and 58.6% for *Block 2*.

The first phase is needed to imitate the performance of the expert-designed system. There is an imbalance among action classes in this training phase in which *Goto+Neck, Intercept+Neck*, and *Block 2* are majority classes which occupy nearly 95% of recorded actions, while *Dash+Neck, Turn+Neck*, and *Neck* are minority classes which only occupy approximately 5% of recorded actions. From this observation, we design two learning scopes to imitate the module for *defensive positioning*: (i) full scope in which all six actions are learned, and (ii) reduced scope in which only majority action classes are learned while minority classes are aggregated into *Block 2* class. We use *Recall* (Equation 13), *Precision* (Equation 14), and *F1 score* (Equation 15), in order to test the classification performance of the final Q-network to recorded demonstrations of states and actions:

(13) $$\begin{array}{l} \text{Recall}=\frac{tp}{tp+fn} \end{array}$$

(14) $$\begin{array}{l} \text{Precision}=\frac{tp}{tp+fp} \end{array}$$

(15) $$\begin{array}{l} \text{F1 Score}=2\times \frac{Precision\times Recall}{Precision+Recall} \end{array}$$

where *tp* denotes true positives, *fn* denotes false negatives, and *fp* denotes false positives. We also use other performance metrics such as *Scored Goals, Conceded Goals*, and *Goal Difference* to evaluate the team performance of the new neural network-based architecture against the benchmark team. The chosen baselines for the latter evaluation purpose are *Baseline 1*, i.e., the performance of the original base team *Gliders2d* against the benchmark team, and *Baseline 2*, i.e., the performance of the base team *Gliders2d*, in which *Block 1* is removed, against the benchmark team.

For the full-scope learning, imitating six actions in the module for *defensive positioning*, the measurements of classification accuracy, provided in Table 1, show poor results for almost all action classes. With the exception of three actions (*Intercept+Neck, Neck*, and *Block 2*), the accuracy measurements are low with F1 score of 0.83053 for *Goto+Neck*, 0.73991 for *Dash+Neck*, and 0.61545 for *Turn+Neck*. This full-scope, six-action based, neural network degrades the performance against the benchmark team. The average score of this implementation versus *YuShan2018* over 16,000 games is denoted by the label *Block 1 6a Imitation* in Table 3. Its goal difference is −0.425, far worse than −0.080 of *Baseline 1*, being worse than −0.413 shown by *Baseline 2* when *Block 1* is deactivated. Clearly, the full-scope imitation learning for all six defense actions in *Block 1* is not effective when the action classes are imbalanced.

**Table 1:** Evaluation results for the Imitation phase of action-value neural networks designed for Block 1's replacement, with general accuracy equal to 0.83317 (where the maximum accuracy is equal to 1.0).

**Metric**	**Goto+Neck**	**Intercept+Neck**	**Dash+Neck**	**Turn+Neck**	**Neck**	**Block 2**
*Recall*	0.79801	0.86169	0.78311	0.59053	0.98606	0.97645
*Precision*	0.84111	0.91208	0.70123	0.64258	0.89791	0.97792
*F1 Score*	0.83053	0.88617	0.73991	0.61545	0.93992	0.97718

**Figure 7:**
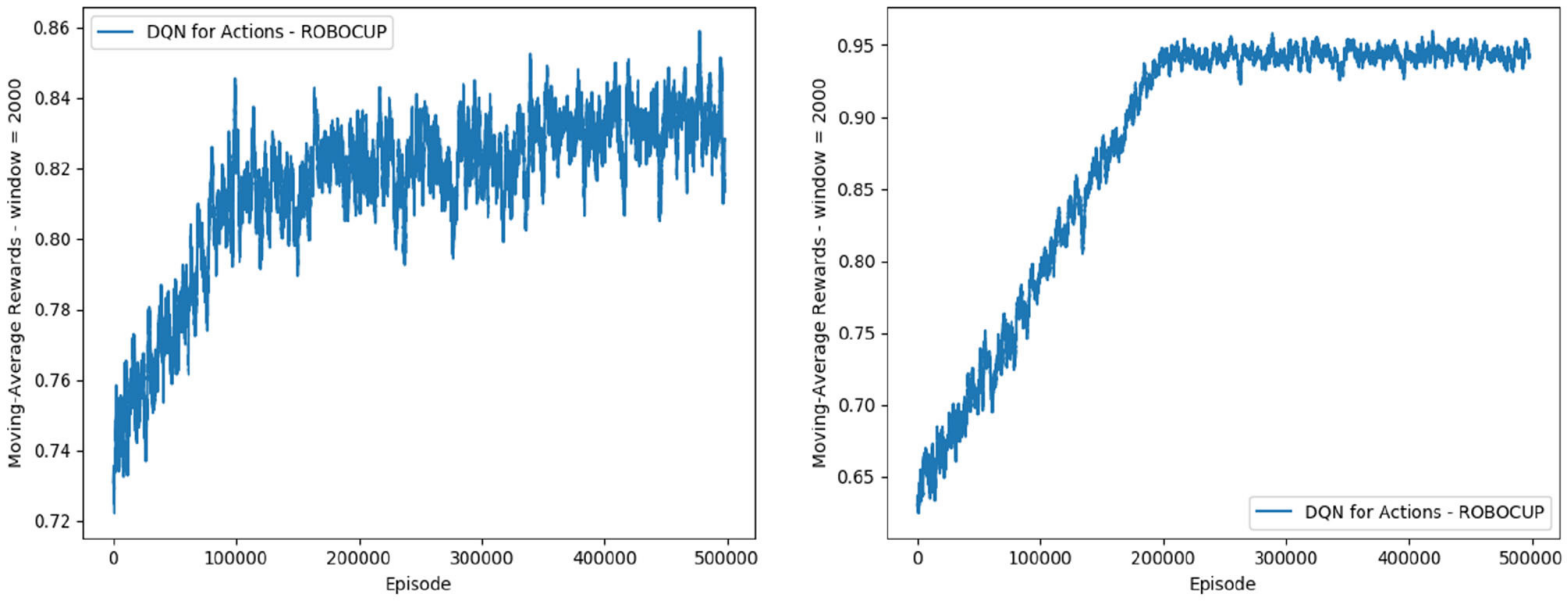
Reward for the case of imitation learning with six action classes (full scope) **(Left)**, and with three action classes (reduced scope) **(Right)**.

For the reduced-scope learning, imitating only the majority action classes in the *defensive positioning* module, we aggregate all minority classes (*Dash+Neck, Turn+Neck*, and *Neck*) within the class *Block 2*. Table 2 shows a significant improvement in terms of the classification accuracy for the two main actions of *Block 1*: F1 score of 0.92727 for *Goto+Neck* (vs. 0.83053 in case of full-scope learning), and F1 score of 0.96324 for *Intercept+Neck* (vs. 0.88617 in case of full-scope learning). With respect to the reward received from the network training with Double DQN algorithm, we also obtain a much more consistent reward curve, traced in the *Right* chart of Figure 7, which may be contrasted with the full-scope learning, traced in the *Left* chart of Figure 7. Finally, considering team performance against the benchmark team, the reduced-scope architecture based on the three-action neural network shows a comparable result (denoted by label *Block 1 3a Imitation* in Table 3), in comparison to *Baseline 1* of the original base team *Gliders2d*. Its goal difference is −0.053 which is slightly better than −0.080 of *Baseline 1*, and much better than −0.413 of *Baseline 2*. These comparisons demonstrate effectiveness of the reduced-scope learning, comprising only three main actions in *Block 1*. The results are checked with Mann-Whitney U test (Table 4), confirming statistically significant differences (see Supplementary Material for more details).

**Table 2:** Evaluation results for Imitation phase of the action-value neural networks designed for Block 1's replacement with three selected actions. The general accuracy is 0.94478 (where the maximum accuracy is equal to 1.0).

**Metric**	**Goto+Neck**	**Intercept+Neck**	**Block 2**
*Recall*	0.95588	0.97298	0.90594
*Precision*	0.90032	0.95369	0.98587
*F1 Score*	0.92727	0.96324	0.94422

**Table 3:** Performance trace for different configurations against the benchmark team, *YuShan*2018. The scores are averaged over 16,000 games for each case.

**Configuration**	**Scored**	**Conceded**	**Goal**	**Std**.	**Winning**	**Drawing**	**losing**
	**goal**	**goal**	**difference**	**error**	**rate (%)**	**rate (%)**	**rate (%)**
Baseline 1	1.030	1.110	−0.080	0.01144	32.97	29.42	37.61
Baseline 2	1.066	1.478	−0.413	0.01222	27.21	25.84	46.96
Block 1 6a Imitation	1.037	1.462	−0.425	0.01211	26.27	26.09	47.64
Block 1 3a Imitation	1.025	1.078	−0.053	0.01135	33.56	29.63	36.81
Block 1 3a 1-05-0	1.060	1.076	−0.016	0.01147	34.69	28.95	36.36
Block 1 3a 1-1-0	1.046	1.075	−0.029	0.01133	34.55	29.24	36.22
Block 1 3a 1-2-0	1.050	1.078	−0.028	0.01125	34.20	29.06	36.74
Block 1 3a 1-1-1	1.043	1.087	−0.044	0.01148	33.93	29.53	36.54
Block 2 2a Imitation	1.026	1.045	−0.019	0.01096	33.35	30.19	36.46
Block 2 2a 1-05-0	1.042	1.038	0.004	0.01084	34.43	29.33	36.24
Block 2 2a 1-1-0	1.040	1.058	−0.017	0.00885	33.42	30.11	36.47
Block 1&2 4a Imitation	1.029	1.186	−0.158	0.01070	31.35	28.56	40.09
Block 1&2 4a 1-05-0	1.043	1.173	−0.130	0.01098	31.66	28.32	40.02
Block 1&2 4a 1-1-0	1.014	1.181	−0.168	0.01105	30.59	28.62	40.79

**Table 4:** Mann-Whitney U test for non-parametric hypotheses with a dataset comprising game scores from 16,000 games for each comparison pair.

	**Scoring**	**Conceding**	**Goal Diff**.	
**Comparison pair**	***p*-value**	***p*-value**	***p*-value**	**Conclusion about significant difference**
Block 1 3a Imitation vs. Baseline 1	0.3472	0.0052	0.0635	*No evidence* for a change in Attacking Skills
				*Strong evidence* for a change in Defending Skills
				*Weak evidence* for a change in Goal Difference
Block 1 3a 1-05-0 vs. Block 1 3a Imitation	0.0018	0.4652	0.0151	*Strong evidence* for a change in Attacking Skills
				*No evidence* for a change in Defending Skills
				*Evidence* for a change in Goal Difference
Block 1 3a 1-1-0 vs. Block 1 3a Imitation	0.0156	0.4873	0.0540	*Evidence* for a change in Attacking Skills
				*No evidence* for a change in Defending Skills
				*Weak evidence* for a change in Goal Difference
Block 1 3a 1-2-0 vs. Block 1 3a Imitation	0.0106	0.3564	0.1122	*Evidence* for a change in Attacking Skills
				*No evidence* for a change in Defending Skills
				*No evidence* for a change in Goal Difference
Block 1 3a 1-1-1 vs. Block 1 3a Imitation	0.0683	0.2697	0.2310	*Weak evidence* for a change in Attacking Skills
				*No evidence* for a change in Defending Skills
				*No evidence* for a change in Goal Difference
Block 2 2a Imitation vs. Block 1 3a 1-05-0	0.0025	0.0205	0.1385	*Strong evidence* for a change in Attacking Skills
				*Evidence* for a change in Defending Skills
				*No evidence* for a change in Goal Difference
Block 2 2a 1-05-0 vs. Block 2 2a Imitation	0.0455	0.3859	0.0579	*Evidence* for a change in Attacking Skills
				*No evidence* for a change in Defending Skills
				*Weak evidence* for a change in Goal Difference
Block 2 2a 1-1-0 vs. Block 2 2a Imitation	0.0661	0.0878	0.4035	*Weak evidence* for a change in Attacking Skills
				*Weak evidence* for a change in Defending Skills
				*No evidence* for a change in Goal Difference
Block 2 2a 1-05-0 vs. Block 1&2 4a Imitation	0.2699	5.467e-25	1.065e-15	*No evidence* for a change in Attacking Skills
				*Strong evidence* for a change in Defending Skills
				*Strong evidence* for a change in Goal Difference
Block 1&2 4a 1-05-0 vs. Block 1&2 4a Imitation	0.0438	0.3201	0.1073	*Evidence* for a change in Attacking Skills
				*No evidence* for a change in Defending Skills
				*No evidence* for a change in Goal Difference
Block 1&2 4a 1-1-0 vs. Block 1&2 4a Imitation	0.1736	0.3883	0.1437	*No evidence* for a change in Attacking Skills
				*No evidence* for a change in Defending Skills
				*No evidence* for a change in Goal Difference

**Figure 8:**
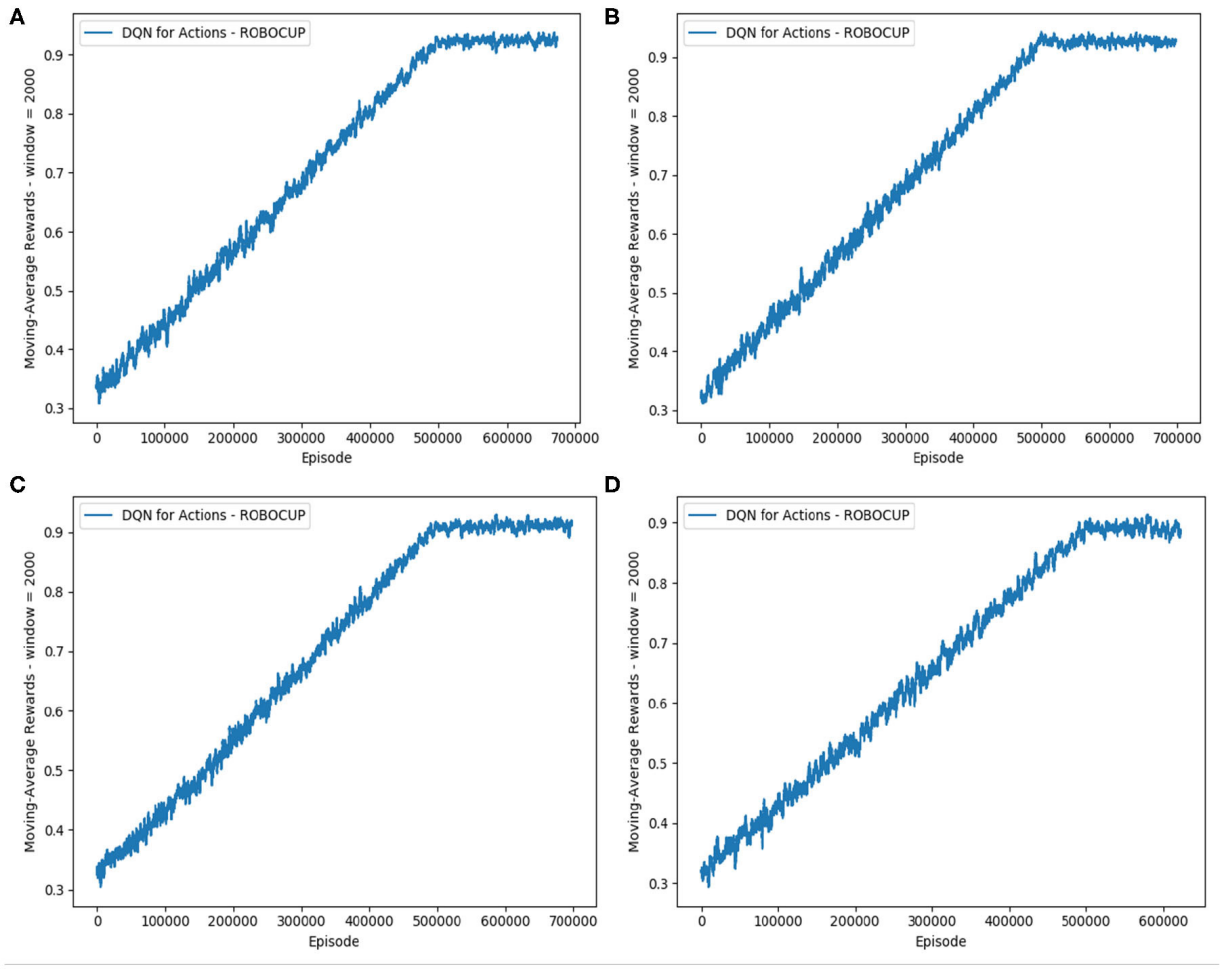
Reward for the cases of the reduced-scope imitation learning with three action classes and introduction of the goal signal. Four different images show different weights of the goal signal. **(A)** λ_1_ = 1 and λ_2_ = 0. **(B)** λ_1_ = 2 and λ_2_ = 0. **(C)** λ_1_ = 0.5 and λ_2_ = 0. **(D)** λ_1_ = 1 and λ_2_ = 1.

In the second phase, we enhance the imitation learning with a delayed reward signal added to the reduced scope with three action classes. The main aim is to improve the team performance beyond the level achieved by the imitation learning alone. Equation (11) shows the integration of the goal signals into the reward function, in order to guide the training process. As in the Figure 8, the curve for the reward received during the training time shows a stable profile of the reduced-scope learning, similar to the case of *Block 1 3a Imitation* traced in Figure 7.

Measuring performance against the benchmark team, we provide the results for the goal signals introduced with different pairs of λ_1_ and λ_2_ (see Equation 11) in Table 3. The notation for configurations summarized in Tables 3, 4 follows the convention that the last 2 indices in the name represent λ_1_ and λ_2_ respectively, and 3*a* represents the reduced-scope imitation learning with three main action classes. For example, *Block 1 3a 1-05-0* represents the reduced-scope learning with λ_1_ = 0.5 and λ_2_ = 0; and *Block 1 3a 1-1-1* represents the reduced-scope learning with λ_1_ = 1 and λ_2_ = 1. Results summarized in Table 3 demonstrate that the goal signal added to the training of Q-networks has improved the team performance. Comparing the team performance with the imitation learning case of *Imitation 3a* vs. the other cases enhanced with the goal signal (*Block 1 3a 1-05-0, Block 1 3a 1-1-0, Block 1 3a 1-2-0*, and *Block 1 3a 1-1-1*), we observe an increase in the goal difference for all the enhanced cases. As shown in Table 4, another interesting effect of a delayed reward signal is an improvement of the attacking skills expressed via the statistically-significant increase in the scored goals across all the cases. However, different configurations of λ_1_ and λ_2_ create different effects resulting from a reward signal. In our experiments, the best configuration *Block 1 3a 1-05-0* is observed with λ_1_ = 0.5 and λ_2_ = 0, with the goal difference increasing from -0.080 (*Baseline 1*) to almost parity against the benchmark, i.e., −0.016, coupled with an improvement in scored goals. Furthermore, the conceded goals' signal seems to have a better effect on training *Estimator* network than a signal based on the scored goals, which is a reasonable outcome of the process tailored to learning defensive behaviors.

**Figure 9:**
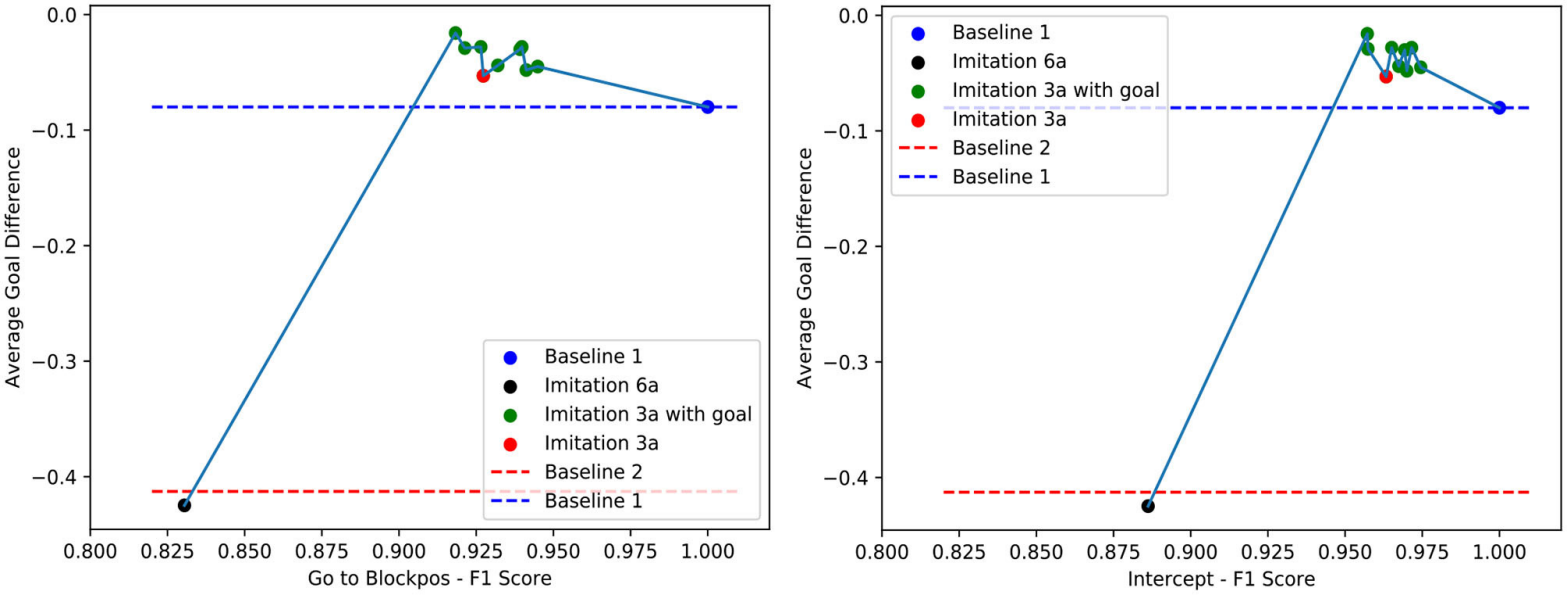
The relation between F1 score of *Goto+Neck* and *Intercept+Neck* in Block 1 replacement task and the average goal difference.

**Figure 10:**
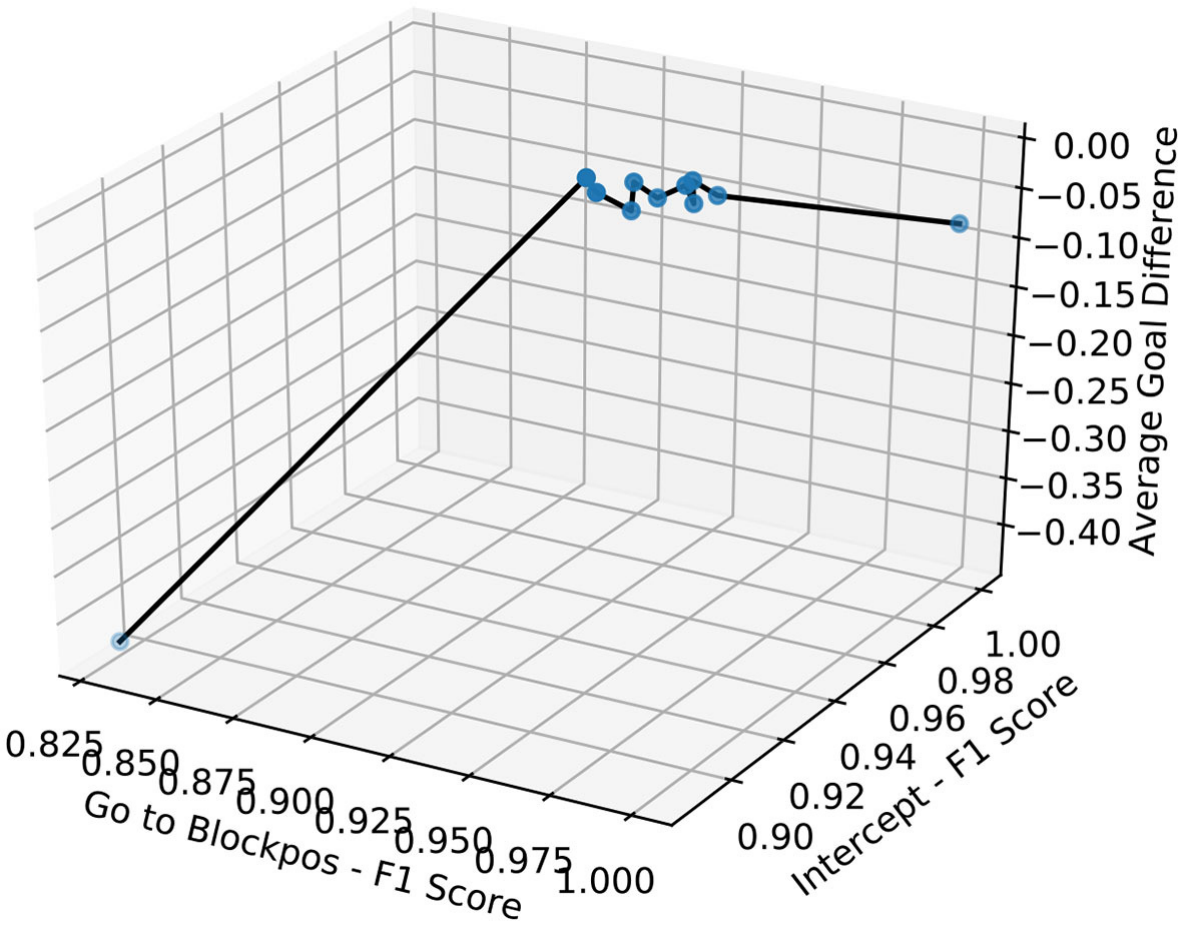
3D plot shows the relation between F1 score of *Goto+Neck* and *Intercept+Neck* in Block 1 replacement task and the average goal difference.

We summarize our experimental results in Figures 9, 10. These graphs show the relation between F1 score and the average goal difference, with the peaks in the two plots highlighting the case of imitation learning with a delayed goal signal (λ_1_ = 0.5 and λ_2_ = 0). Although the improvements resulting from the addition of delayed reward signal to the learning can be observed in almost all cases, the variation in the average goal difference again implies that the performance of the system depends on the selection of λ_1_ and λ_2_.

### 4.2. Replacement of Block 1 and Block 2 With RL-Based Neural Networks With Original Structure Preserved

In previous section, we reported a consistent and positive impact of the goal signal added to the training of the action-based neural network, which replaced *Block 1*. Now we apply the same approach to imitate *Block 2* to demonstrate the applicability of the RL-based training framework in the two cases: (i) to imitate two action classes in *Block 2*, and (ii) to imitate these action classes with the conceded goals' signal introduced to the Q-networks training. The training process was performed using a new training dataset comprising the records from over nineteen million defensive actions (7.4% is *Pressing* and 92.6% is *Others*) in *Block 2* from 10,000 games of our best team so far, *Block 1 3a 1-05-0*, playing against team *YuShan*2018. The trained Q-network which replaced *Block 2* is then processed in sequence, following the Q-network which replaced *Block 1*. The performance results are shown in Tables 3, 4, with the notation following the naming convention where 2*a* denotes the two action classes in *Block 2*, and the last two indices representing the value selection for λ_1_ and λ_2_ in the reward function, see Equation 11.

Considering the first imitation learning case, we note that the performance of the newly learned network for *Block 2*, denoted by *Block 2 2a Imitation*, is comparable to that of *Block 1 3a 1-05-0*. This is evidenced by a parity in the goal difference between these configurations, shown in Table 3 and confirmed by no evidence for a change in Goal Difference reported for the case of *Block 2 2a 1-05-0 vs. Block 2 2a Imitation* in Table 4.

The results of the second imitation learning case with a delayed reward signal are presented in Tables 3, 4. These results demonstrate a positive effect of this reward signal on the attacking skills of the agent. Mann–Whitney *U*-test shows the change in the scored goals with *p*-value=0.0455 for the case of *Block 2 2a 1-05-0* (*Evidence for a change in Attacking Skills*) and *p*-value=0.0661 for the case of *Block 2 2a 1-1-0* (*Weak evidence for a change in Attacking Skills*). This inference is similar to the one drawn from the replacement of *Block 1* in section 4.1. With the introduction of the delayed reward signal, we observe an improvement in *Goal Difference* with respect to the pure imitation learning case. The configuration with λ_1_ = 0.5 and λ_2_ = 0, *Block 2 2a 1-05-0*, shows a highest goal difference observed in our experiments at 0.004, which is statistically confirmed as a significant difference, see Table 4. Thus, the effectiveness of the proposed learning framework is shown for the full case extended over the original structure of the expert-designed code incorporating both *Block 1* and *Block 2*.

### 4.3. Replacement of Block 1 and Block 2 With RL-Based Neural Networks and Without Structure Preservation

In section 4.2, the original expert-designed structure is retained with the sequential order of two action-value networks replacing *Block 1* and *Block 2*. In this section, we study another question, considering whether the structure of a well-optimized expert-designed system is worth preserving. We set up a new experiment with a single neural network which learns all actions in both *Block 1* and *Block 2*. Performance of the agent using this network represents the end-to-end approach, and is used to compare with the case *Block 2 2a 1-05-0* in which a system of two neural networks in a preserved sequence replaces the original code for *Block 1* and *Block 2*. The training process was performed using a new training dataset comprising over fifteen million records from all defensive action classes (6.6% for *Goto+Neck*, 1.4% for *Intercept+Neck*, 12.1% for *Pressing*, and 79.9% for *Others*) of the team *Block 2 2a 1-05-0*, against the benchmark team *YuShan*2018.

Unlike the results presented in sections 4.1 and 4.2, the team performance summarized in Table 3 for the pure imitation case, *Block 1*&*2 4a Imitation*, shows a significant degradation with respect to the previous baseline, *Block 2 2a 1-05-0*. Using statistical test results shown in Table 4, we observe strong evidence for a negative shift in the goal difference. This outcome mainly stems from weakened defensive skills, a conclusion confirmed by the comparison pair *Block 2 2a 1-05-0 vs. Block 1*&*2 4a Imitation*. Interestingly, the effect of adding the delayed reward signal on the team performance is still marginally positive, as observed in the case of λ_1_ = 0.5 and λ_2_ = 0 with an approximate increment of 0.028 in goal difference. This is further confirmed by results shown in Table 4. With respect to the goal difference, we confirm that there is a very weak evidence for a change in this measurement, as the p-value for the comparison pair “*Block 1*&*2 4a 1-05-0 vs. Block 1*&*2 4a Imitation”* is 0.1073, being very close to the limit α = 0.1.

### 4.4. Correlation Between Imitation Learning and Performance

We have developed and investigated an RL-based neural network replacing the *defensive positioning* module in the base team *Gliders2d*. Both the empirical evidence (the average performance over 16,000 games) and statistical hypothesis testing using Mann–Whitney *U*-test, show the effectiveness of a delayed reward signal, i.e., *delayed goal signal*, added to the training of action-value neural networks. This is demonstrated for both cases: (i) when the existing structure of the original behavioral selection is preserved, see section 4.1 and section 4.2, or (ii) when the existing structure is not preserved, see section 4.3.

Importantly, we observe a clear degradation in team performance when the action policy learning is carried out without preserving the structure of the original well-optimized expert-designed system. Without the sequential order of Block 1 and Block 2, the final single neural network constructs a single probabilistic “*black box”* aiming to make a decision across all of the actions in *Block 1* and *Block 2*. However, the resultant statistically-significant poor performance of “flattened” structure, summarized in Tables 3, 4 with the prefix “*Block 1*&*2”*, implies a clear benefit of preserving the structure of a well-optimized expert-designed system.

## 5. Conclusion

We introduced and evaluated an RL-based framework extended with a delayed reward signal. This framework successfully learned a range of behaviors by imitating an action policy produced by an expert-designed system, and improved beyond this baseline level of performance. This approach was designed and evaluated using the multi-agent environment offered by the RoboCup Simulation League, with a specific focus on improving *defensive positioning* provided by the base code of *Gliders2d*. The overall improvement in team performance was extensively tested for statistical significance, confirming effectiveness of the proposed RL-based learning scheme in the context of multi-agent environment of RoboCup.

In doing so, we categorized and grouped some actions within a minority class, used in the initial training. Specifically, in learning defensive behavior, some actions which play a relatively minor role were excluded from the training. This approach has a limited general applicability. For example, in implementing the shooting action which plays a critical role, such minority classes would be evaluated under a different hierarchical scheme: either by human experts (using a coded hierarchy) or by a higher-level action policy decider. An example of such implementation is given by MaxQ algorithm (Bai et al., 2015).

In addition, we contrasted the learning architectures with and without preservation of an underlying expert-designed structure. This demonstrated a clear utility of a structure-preserving neural architecture when dealing with highly optimized expert solutions. This observation may appear to contradict other examples where the end-to-end learning has been found to offer advantages over machine learning solutions using a human-defined structure (Lecun et al., 2006; Collobert et al., 2011; Mnih et al., 2013; Bojarski et al., 2016). However, the specific problem of learning defensive behaviors considered in this paper was affected by a strong prior optimization within a well-defined structure of *Gliders2d*, a baseline agent code used by the world champion teams *Gliders* and *Fractals* (Prokopenko and Wang, 2019a,b).

In general, our results may have a broad appeal, emphasizing a continuing importance of the *divide-and-conquer* approaches to complex problems (Glasmachers, 2017). The structure-preserving imitation learning augmented by delayed rewards is likely to find applications in multi-agent cooperation and collective behavior (Prokopenko and Wang, 2004; Xu et al., 2013; Bai et al., 2015; Hamann et al., 2016; Cliff et al., 2017), modular robotics (Prokopenko et al., 2006; Martius et al., 2007; Der and Martius, 2012), and distributed networks and dynamical systems in general (Mortveit and Reidys, 2007; Cliff et al., 2013, 2016; Hefny et al., 2015).

## Data Availability Statement

The original contributions presented in the study are included in the article/Supplementary Material, further inquiries can be directed to the corresponding author/s.

## Author Contributions

MP conceived and supervised the study. QN developed the theoretical formalism, designed solutions, and performed numerical simulations. All authors contributed to the article and approved the submitted version.

## Conflict of Interest

The authors declare that the research was conducted in the absence of any commercial or financial relationships that could be construed as a potential conflict of interest.
