# Supplementary material for: Structure-Preserving Imitation Learning With Delayed Reward: An Evaluation Within the RoboCup Soccer 2D Simulation Environment
Source: Front Robot AI. 2020 Sep 16;7:123. doi: 10.3389/frobt.2020.00123 (PMC7805756; doi:10.3389/frobt.2020.00123)
Supplement: Supplementary file 1 [file Presentation_1.pdf]

frontiers

Supplementary Material

1 LEARNING ALGORITHM

Algorithm 1: Imitation Learning with Delayed Reward Signal

Input: Demonstrations D recorded from multiple soccer games when defensive behavioral module is activated in all soccer agents. Demonstrations from a game are not necessarily consecutive. Information about goal cycles.

Output: Neural-network-based action policy learned from demonstrations.

Pre-learning Steps:

Generate goal delayed reward ($r_{goal,t}$) to recorded demonstrations following Equation 11. This artificial and back-propagated (*delayed*) signal, obtained from actual scored/conceded goals in the future cycles, is generated for *every* transition.

Link demonstrations in a sequence.

Build *Offline Learning Environment (OLE)* for RL-based imitation learning. OLE can reset its initial state at any transition, and finish each episode at the end of corresponding sequence.

Learning Procedure:

for $i = 1$ to *maximum episodes* **do**
  Reset OLE and receive initial state s_0 containing agent index and state information.
  while episode i does not end **do**
    Select action $a_t = \arg\max_a(\{Q(s_t, a\})$ with ϵ-greedy exploration.
    Receive r_t, s_{t+1}, and $done$ signal from OLE based on Equation 11 and 12.
    Store (s_t, a_t, r_t, s_{t+1}) in *replay buffer*.
    if *replay buffer* size reaches threshold N_r **do**
      Sample a random minibatch of transitions with size N_m from *replay buffer*.
      Update $Estimator$ network parameters θ using standard loss in Equation 7 and 8.
      For all K episodes, update *Target* network parameter $\theta^{'}$ by equation:

$$\theta^{'} \leftarrow \tau\theta + (1-\tau)\theta^{'}_{i}$$

    end if
  end while
end for

We use a network structure with two ReLU-activated hidden layers of 400 and 300 nodes for *Estimator* and *Target* network. They are trained by Adam optimizer (Kingma and Ba, 2014) with the rate linearly-decreased learning from 0.005 over the updating steps. Replay buffer is kept at maximum 4×10^6 transitions, and is isolated from the initial demonstration set with bootstrapping feature enabled (i.e., may contains repeated transitions). N_r is set at minimum 1×10^6 to start the network update. Minibatch size N_m is chosen at 1024 transitions. K is set at 100, and τ is 0.01 to update *Target* network. In Evaluation phase, network parameters of *Estimator* are extracted and embedded within *defensive positioning* module to test the new team's performance.

2 EVALUATION METHOD

In order to evaluate the performance of a soccer team in a highly uncertain non-deterministic environment of RCSS, we need to play a significant number of games to reduce the effect of noise. As reported by Bai et al. (2013), 100 games were played to obtain average goals, average points, and winning rate of team WrightEagle against four other teams with an error rate from 5.6% to 8.8%. In Budden et al. (2015), average goal difference over 1,000 games was used to rank the teams participated in RoboCup 2012. Other studies, e.g., Prokopenko and Wang (2019b) and Prokopenko and Wang (2019a), used between 2,000 and 16,000 games required to achieve a more accurate estimation of the average goal difference, with standard error of the mean (SEM) in the order of 0.01 to 0.05. In this paper, we extend the metric set to {*scoring goals, conceding goals, average goal difference, winning rate, drawing rate, losing rate*}. The evaluation experiments described in our study utilized up to 16,000 games.

We consider game scores of different modified versions of *Gliders2d* as different sample sets. Specifically, *Gliders1* and *Gliders2* are two modified versions of *Gliders2d*, using different neural networks' parameter sets. The sets R_1 and R_2 capture the corresponding game results against a benchmark team *Yushan2018* (Cheng et al., 2018). The statistical hypothesis tests include:

$$H_0 : \textit{Samples from } R_1 \textit{ and } R_2 \textit{ have the same distribution.}$$

$$H_1 : \textit{Samples from } R_1 \textit{ and } R_2 \textit{ have different distributions.}$$

If H_0 is not rejected, it means that statistically the team performances of *Gliders1* and *Gliders2* against *Yushan2018* are the same. On the other hand, if H_0 is rejected, it means that statistically, the team performances of *Gliders1* and *Gliders2* against the benchmark team are different. Then we can use the average game scores to make conclusion about the improvement or degradation of $Gliders1$ in comparison with $Gliders2$.

Since the statistics of the population of R_1 and R_2 are not specified and the game results are independent of each other, we model this test as a nonparametric statistical hypothesis test and apply Mann-Whitney U test method (Mann and Whitney, 1947). The significance level α is selected at three levels of 0.1, 0.05, and 0.01 with different conclusions as follows:

- If p-value in the range $(1, 0.1]$, there is no evidence to reject H_0.
- If p-value in the range $(0.1, 0.05]$, there is weak evidence to reject H_0.
- If p-value in the range $(0.05, 0.01]$, there is evidence to reject H_0.
- If p-value in the range $(0.01, 0)$, there is strong evidence to reject H_0.

REFERENCES

Bai, A., Wu, F., and Chen, X. (2013). Towards a principled solution to simulated robot soccer. In *RoboCup 2012: Robot Soccer World Cup XVI* (Springer, Berlin, Heidelberg), vol. 7500

Budden, D., Wang, P., Obst, O., and Prokopenko, M. (2015). Robocup simulation leagues: Enabling replicable and robust investigation of complex robotic systems. *IEEE Robotics and Automation Magazine* 22, 140–146. doi:10.1109/MRA.2015.2446911

Cheng, Z., Xie, N., Sun, C., Tan, C., Zhang, K., Wang, L., et al. (2018). Yushan2018 team description paper for RoboCup2018. In *RoboCup 2018: Robot World Cup XXII* (Montreal, Canada)

Kingma, D. P. and Ba, J. (2014). Adam: A method for stochastic optimization. *CoRR* abs/1412.6980

Mann, H. and Whitney, D. (1947). On a test of whether one of two random variables is stochastically larger than the other. *Annals of Mathematical Statistics* 18, 50–60

Prokopenko, M. and Wang, P. (2019a). Fractals2019: Combinatorial optimisation with dynamic constraint annealing. In *RoboCup 2019: Robot World Cup XXIII*, eds. S. Chalup, T. Niemueller, J. Suthakorn, and M.-A. Williams (Cham: Springer International Publishing), 616–630

Prokopenko, M. and Wang, P. (2019b). Gliders2d: Source Code Base for RoboCup 2D Soccer Simulation League. In *RoboCup 2019: Robot World Cup XXIII*, eds. S. Chalup, T. Niemueller, J. Suthakorn, and M.-A. Williams (Cham: Springer International Publishing), 418–428
